# Effect of kaolinite edge surfaces on formation of Tb^3+^-doped phosphor by solid-state reaction†

**DOI:** 10.1039/d2ra02199d

**Published:** 2022-05-20

**Authors:** Shingo Machida, Ken-ichi Katsumata, Atsuo Yasumori

**Affiliations:** Department of Material Science and Technology, Faculty of Advanced Engineering, Tokyo University of Science 6-3-1 Niijuku, Katsushika-ku Tokyo 125-8585 Japan shingo.machida@rs.tus.ac.jp

## Abstract

This study assessed the effect of kaolinite edge surfaces on solid-state reactions. Specifically, Tb^3+^-doped metastable CaAl_2_Si_2_O_8_ showing green phosphorescence was prepared *via* a solid-state reaction between expanded kaolinite, a methoxy-modified kaolinite, having Tb^3+^ ions adsorbed on its edge surfaces and CaCO_3_. This material cannot be obtained by the conventional grinding of kaolinite, CaCO_3_ and Tb_2_O_3_, indicating that the use of kaolinite edge surfaces is advantageous as a means of achieving certain solid-state reactions.

## Abstract 2

The effect of kaolinite edge surfaces on a solid-state reaction was demonstrated by the synthesis of Tb^3+^-doped metastable CaAl_2_Si_2_O_8_, indicating the importance of Tb^3+^ adsorbed on kaolinite edge surfaces.

## Graphical abstract

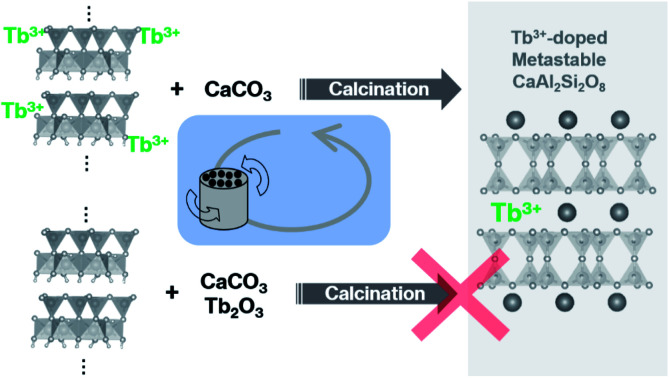

## Introduction

The grinding of two or more raw materials that have been mixed together is known to promote various solid-state reactions that can generate numerous inorganic solid products.^1,2^ This grinding procedure also disrupts the stacking order of layered inorganic solids.^3–9^ In previous report by our group using expanded kaolinite as a raw material, this disordering process was facilitated by layer expansion, resulting in the rapid formation of target products.^9^ Kaolinite is a layered clay mineral having the formula Al_2_Si_2_O_5_(OH)_4_ and, while each layer in the kaolinite structure is neutral, each edge represents a crystal fracture surface bearing cation exchange sites.^10,11^ The number of available kaolinite edges was also found to increase as a result of layer expansion in our previous study, and so we are interested in the possibility of using these edges to promote solid-state reactions.^12^

## Experimental

### Solid-state reaction

The present study examined the solid-state reactions of kaolinite on which Tb^3+^ was adsorbed in conjunction with grinding with calcium carbonate (CaCO_3_) to form Tb^3+^-doped metastable CaAl_2_Si_2_O_8_. This compound is a member of the layered materials having the formula RAl_2_Si_2_O_8_ where R is an alkaline earth metal ion.^13^ The raw materials and grinding conditions used in this research were identical to those employed in our previous study.^9^ The kaolinite raw material was expanded Kanpaku kaolinite (JCSS-1101c, obtained from the Clay Science Society of Japan) without impurities (referred to herein as Ex-Kaol), a methoxy-modified kaolinite.^9^ This material was used to rapidly generate metastable CaAl_2_Si_2_O_8_ containing minimal byproducts (referred to herein as m-CAS).^9^ All the chemicals used in this study were reagent grade. In each reaction, a quantity of the kaolinite products was combined with CaCO_3_ (Hayashi Pure Chemical Ind., Ltd.) in a 1 : 1 molar ratio and roughly mixed using an agate mortar and pestle. A 358 mg portion of the resulting solid was subsequently dispersed in methanol (8 mL) and milled in a planetary ball mill at 250 rpm for 12 h using a resin vessel (12.5 mL) and 120 silicon carbide balls (2.5 mm in diameter). After milling, the solid was separated by centrifugation at 5000 rpm for 1 min and then dried at 80 °C for 1 h, to provide the product referred to herein as the “ground raw material”. The calcination of this material was performed by heating at 900 °C for 4.5 h. The majority of the calcinations in this study were conducted under air at a heating rate of 10 °C min^−1^ with subsequent furnace cooling.

### Solid-state reactions including Tb^3+^ or Tb_2_O_3_

A portion of the ground raw material (125 mg) generated using the process described above was immersed in 5 mL of an aqueous solution containing 0.50 mol L^−1^ terbium chloride (TbCl_3_, Wako Pure Chemical) for 24 h. The solid was subsequently recovered by centrifugation, washed with distilled water to remove chloride ions (as determined by adding a silver nitrate solution to the supernatant) and dried at 80 °C for 24 h. The resulting material was calcined at 900 °C for 12 h (referred to herein as m-CAS-Tb-Edge). For comparison purposes, Ex-Kaol was also mixed with CaCO_3_ containing either 0.3 or 0.075 mol% Terbium oxide (Tb_2_O_3_, Kojundo Chemical Laboratory) and subjected to the same grinding procedure described above, followed by calcination at 900 °C for 12 h. These products are designated herein as m-CAS-Tb-X, where X is the Tb_2_O_3_ content. When producing these specimens, the CaCO_3_ amount was decreased to offset the increase in Tb_2_O_3_. In addition, a portion of the same ground raw material used to produce the m-CAS-Tb-0.3 was instead subjected to a 900 °C calcination for 4.5 h (referred to herein as m-CAS-Tb-0.3-4.5 h). A reference green phosphor specimen was obtained by performing, a solid-state reaction between the Ex-Kaol and barium carbonate (BaCO_3_, Wako Pure Chemical) together with 0.3 mol% Tb_2_O_3_ to form Tb^3+^-doped BaAl_2_Si_2_O_8_ (BAS-Tb-0.3), based on a procedure reported in a previous paper.^8^ This sample was processed using similar grinding conditions to those employed in generating the m-CAS and was calcined at 1100 °C for 12 h.^8^ Notably, layered BaAl_2_Si_2_O_8_ has previously been used to synthesize a Tb^3+^-doped phosphorescent material.^14^

### Solid-state reactions including Ca^2+^ or a reducing atmosphere

The reaction mechanism in this work was examined by performing, the same immersion procedure described above but using 125 mg of the ground raw material and 5 mL of an aqueous solution containing 0.50 mol L^−1^ calcium chloride (CaCl_2_, Wako Pure Chemical). The resulting solid was then calcined at 900 °C for 4.5 h (referred to herein as m-CAS-Ca-Edge). In addition, a portion of the ground raw materials used to produce the m-CAS were calcined at 900 °C for 4.5 h under a nitrogen containing 3 vol% hydrogen gas to provide a reducing environment, producing a specimen referred to herein as m-CAS-R.

### Characterization

The crystalline phases and particle morphologies of the various materials were characterized by acquiring X-ray diffraction (XRD) patterns (XRD-6100, Shimadzu) at a scan rate of 1° min^−1^ and by field-emission scanning electron microscopy (FE-SEM, spra40, Zeiss), respectively. Prior to obtaining FE-SEM images, samples were sputter-coated with platinum. The Tb concentration in the m-CAS-Tb-Edge (with the Tb present in the form of Tb_2_O_3_) was assumed to equal that in the raw material before calcination, which was estimated to be 0.12 mol% in the material based on inductively coupled plasma (ICP) analysis (ICPE-9000, Shimadzu). Prior to this analysis, a 50 mg portion of the ground raw material (comprising Ex-Kaol and CaCO_3_) that had been immerse in the Tb^3+^ solution was dispersed in a 1 mol L^−1^ hydrochloric acid (5 mL), and stirred for 1 h. Following this, the product was recovered by centrifugation at 5000 rpm for 5 min, and then washed twice, each time with 10 mL of the same hydrochloric acid solution. All the supernatants were mixed and appropriately diluted prior to assessment by ICP. The product colourations were also assessed by visible-light reflectance spectroscopy (v-670 with an ARSN-733 attachment, JASCO). Prior to these analyses, samples were mixed with barium sulfate (BaSO_4_, Wako Pure Chemical). The luminescence of each product was characterized by fluorescence spectroscopy (FP-6500 with an integrating sphere unit, ISF-513, JASCO) with excitation at 376 nm.^14^ The valence state of Tb ion was characterized by X-ray photoelectron spectroscopy (XPS; JPS-9030, JEOL).

## Results and discussion

Photographic images showing the colour of a specimen under 254 nm irradiation before and after the acid treatment are provided in Fig. S1.† These images indicate the disappearance of the green phosphorescence under 254 nm irradiation following exposure to acid. This disappearance and the cause of the weak green phosphorescence obtained from the powder before the acid treatment are discussed further.

**Fig. 1:**
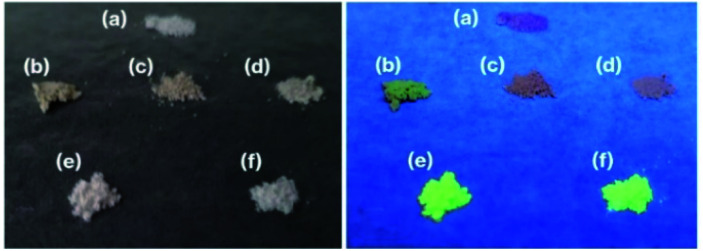
Photographic images of (a) m-CAS, (b) Tb_2_O_3_, (c) m-CAS-Tb-0.3, (d) m-CAS-Tb-0.075, (e) m-CAS-Tb-Edge, and (f) BAS-Tb-0.3 acquired under (left) normal lighting and (right) 254 nm irradiation.

**Fig. 2:**
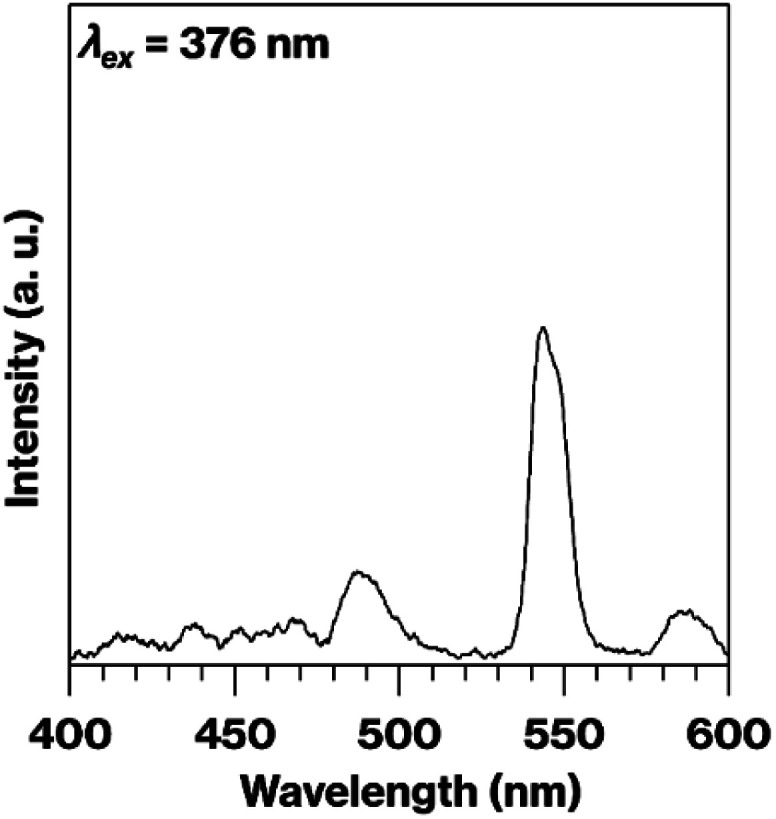
Fluorescence spectrum of the m-CAS-Tb-Edge.

Fig. 1 presents photographic images of the various solid products. The m-CAS had a white colouration (Fig. 1a), in agreement with the previous report.^9^ Interestingly, this white colour was not obtained when Ex-Kaol was not used as the raw material.^9^ In contrast to the m-CAS, the m-CAS-Tb-0.3 and −0.075 were light brown and thus had a similar colour to that of Tb_2_O_3_ (Fig. 1b–d). The m-CAS-Tb-Edge was white and was found to emit intense green phosphorescence upon excitation at 254 nm (Fig. 1e). This behaviour matched that of the BAS-Tb-0.3 (Fig. 1f), which also exhibited green phosphoresence.^14^ The fluorescence spectrum of the m-CAS-Tb-Edge (Fig. 2) is typical for Tb^3+^ ions, with green emission due to the ^5^D_4_ → ^7^F_5_ transition at 543 nm.^14^ This emission is absent in the spectra of the m-CAS and m-CAS-Tb-0.075 (data not shown). The product colourations observed under normal lighting were in agreement with the visible-light spectra shown in Fig. S2.† The absorption at relatively short wavelengths evident in the m-CAS and m-CAS-Tb-Edge spectra is attributed to the presence of silicate layers (Fig. S2a and b†).^15,16^ Compared with the m-CAS and m-CAS-Tb-Edge, the m-CAS-Tb-0.075 and -0.3 as well as the Tb_2_O_3_ all exhibited increased absorption in the shorter wavelength region (Fig. S2c–e†). Specifically, the spectrum of each contained a shoulder at approximately 450 nm. This was likely due to the presence of Tb^4+^ ions, which were found to produce darker sample colours in a prior study.^17^ It should be noted that the oxidation of Tb^3+^ to Tb^4+^ is known to be responsible for the change in the colour of terbium oxide from white (Tb_2_O_3_) to dark brown (Tb_7_O_12_ or Tb_11_O_20_).^18,19^ In the present study, the Tb_2_O_3_ was originally light brown (Fig. 1b). The XPS spectrum of the Tb_2_O_3_ used in this study as presented in Fig. S3† displayed a broad signal that was deconvoluted into peaks at 156.5 and 150.5 eV, corresponding to Tb^4+^ and Tb^3+^, respectively.^20^ Therefore, this material contained Tb^4+^ and, based on the respective peak integrals, the Tb^4+^/Tb^3+^ molar ratio was estimated at 0.22. When Tb_2_O_3_ is heated in air, the original TbO_1.5_ (Tb_2_O_3_) compound is oxidized to TbO_1.714_ (Tb_7_O_12_) in the 500–900 °C range, while TbO_1.714_ (Tb_7_O_12_) is reduced back to TbO_1.5_ (Tb_2_O_3_) above 1000 °C.^19^

Fig. S4† shows FE-SEM images of the raw materials and the calcined products. The Ex-Kaol was evidently composed of hexagonal plate-like particles (Fig. S4a†)^12^ while the CaCO_3_ comprised cubic or rhombus-like particles with various sizes (Fig. S4b†). The m-CAS, m-CAS-Tb-0.3 and m-CAS-Tb-Edge (Fig. S4d and e†) had different morphologies from those of the Ex-Kaol, CaCO_3_ and Tb_2_O_3_ (Fig. S4a–c†), and contained hexagonal plate or rhombus-like particles with sizes on the order of 500 nm. In addition, the images of those calcined products are similarly (Fig. S4d and e†). It is noteworthy that metastable-CaAl_2_Si_2_O_8_ is composed of hexagonal plate-like particles.^21,22^

**Fig. 3:**
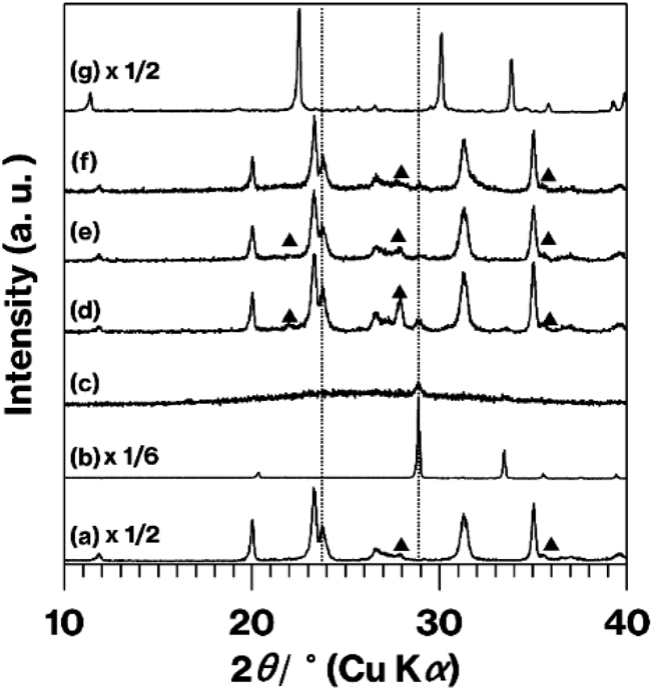
XRD patterns for (a) m-CAS, (b) Tb_2_O_3_, (c) m-CAS-Tb-0.3-4.5 h, (d) m-CAS-Tb-0.3, (e) m-CAS-Tb-0.075, (f) m-CAS-Tb-Edge, and (g) BAS-Tb-0.3. Filled triangles indicate anorthite reflections. Some of these profiles have been vertically compressed to allow easier comparison.

**Fig. 4:**
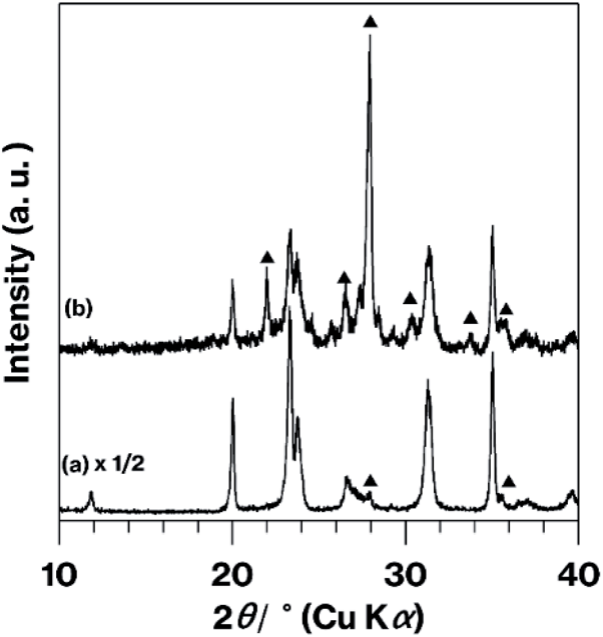
XRD patterns for (a) m-CAS and (b) m-CAS-Ca-Edge. Filled triangles indicate anorthite reflections. The bottom profile has been vertically compressed to allow easier comparison.

Fig. 3 presents XRD patterns obtained from the various products and from pure Tb_2_O_3_. The m-CAS pattern (Fig. 3a) shown here matches that reported previously.^9^ The intense reflection at 28.9° (two theta) that appears in the Tb_2_O_3_ pattern (Fig. 3b) is also present in the patterns for the CAS-based products (Fig. 3c–f) but was not generated by BAS-Tb-0.3 (Fig. 3g), which produced a pattern similar to those previously published in the literature.^14^ The m-CAS-Tb-0.3-4.5 h primarily produced a halo pattern (Fig. 3c). In addition, the reflections observed in the m-CAS pattern are present in those obtained from the other CAS-based products (Fig. 3d–f). None of the patterns generated by the CAS products contained any reflections due to CaCO_3_, Ex-Kaol, or the raw ground material, as demonstrated by Fig. S5.† Of note, the pattern for the CaCO_3_ used in this study displayed reflections due to calcite,^23^ while that for Ex-Kaol exhibited 0.86 and 0.72 nm diffraction lines resulting from the basal spacings of Ex-Kaol and pristine kaolinite, respectively, in agreement with a previous report.^9^ In particular, the 0.72 nm diffraction line is commonly observed in studies of kaolinite intercalation.^5^ The intensity of the reflection due to Tb_2_O_3_ (2*θ* = 28.9°) relative to the intensity of the m-CAS reflections increases in the order of m-CAS-Tb-0.3, m-CAS-Tb-Edge and m-CAS-Tb-0.075, which is consistent with the relative Tb_2_O_3_ concentrations in these materials of 0.30, 0.12 and 0.075 mol%, respectively. In addition, the relative intensities of the reflections due to anorthite (the stable phase of CaAl_2_Si_2_O_8_)^7,9^ decrease in the same order. Notably, the position (2*θ* = 23.8°) of the (004) reflection^21^ due to the stacking direction of the aluminosilicate layers of metastable CaAl_2_Si_2_O_8_ remains constant in the patterns for the CAS-based products (Fig. 3a and d-f). It is also evident that more intense anorthite reflections were generated by the m-CAS-Ca-Edge compared with the m-CAS (Fig. 4). In previously published XRD patterns for Tb_2_O_3_ containing Tb^4+^,^18,19^ the Tb_2_O_3_ reflections became broader with increasing the Tb^4+^ concentration. The reflection due to Tb_2_O_3_ observed in the XRD pattern for the Tb_2_O_3_ used in this study (Fig. 3b) has the same general shape in these prior patterns.^18,19^

Based on the formulae for the various terbium oxides, more than half of the Tb in TbO_1.714_ (Tb_7_O_12_) is in the form of Tb^3+^, while the more oxidized state of TbO_1.75_ (Tb_4_O_7_) contains equivalent amounts of Tb^3+^ and Tb^4+^. Taking the light brown colouration, visible-light spectra, XPS spectrum, and XRD patterns of the m-CAS-Tb-0.30 and m-CAS-Tb-0.075 (Fig. 1c, d, S2c, e,† and 3b–f) into consideration, these products likely contained partially oxidized Tb_2_O_3_. In contrast, the m-CAS-Tb-Edge was a white powder (Fig. 1e) with a Tb_2_O_3_ concentration of 0.12 mol%. In a previous study, Tb_2_O_3_ was formed by heat treatment of Tb(OH)_3_.^24^ It is well-known that rare-earth ions typically form inner and outer sphere complexes^25,26^ surrounded by water molecules on kaolinite edge and layer surfaces, respectively.^27,28^ In addition, the TbCl_3_ removed upon washing in the present study was converted into Tb_7_O_12_ by the heat treatment.^29^

The data presented herein demonstrate that the m-CAS-Tb-Edge exhibited green phosphorescence (Fig. 1f and 2) as a consequence of immersing the ground raw materials in an aqueous solution of Tb^3+^. It should be noted that this light emission could not be obtained when using a conventional grinding procedure (Fig. 1b–d and 3c–e), indicating that Tb^3+^ ions were not readily incorporated in the metastable CaAl_2_Si_2_O_8_ based on the solid-state reactions of Ex-Kaol, CaCO_3_ and Tb_2_O_3_. In contrast, the green phosphor BAS-Tb-0.3 forms as described in a previous report.^14^ In the present study, the metastable CaAl_2_Si_2_O_8_ and layered BaAl_2_Si_2_O_8_ were calcined at 900 and 1100 °C, respectively. The formulas for terbium oxide could be TbO_1.714_ (Tb_7_O_12_) and TbO_1.5_ (Tb_2_O_3_) at these respective temperatures.^19^ Although more than 50% of the Tb in the former material is in the form of Tb^3+^, the presence of Tb^4+^ likely interfered with the incorporation of Tb^3+^ in the metastable CaAl_2_Si_2_O_8_. According to Shannon,^30^ the ionic radii of Ba^2+^ (0.142 nm) and Ca^2+^ (0.110 nm) are larger than those of Tb^3+^ (0.104 nm) and Tb^4+^ (0.88 nm). The larger ionic radius of Ba^2+^ compared with Ca^2+^ implies that the basal spacing of metastable CaAl_2_Si_2_O_8_ is smaller than that of layered BaAl_2_Si_2_O_8_.^14^ Also, the differences in ionic radius between Ca^2+^ ions and Tb ions indicates that the basal spacing of metastable CaAl_2_Si_2_O_8_ is insufficient to incorporate Tb^3+^ ions. Even so, the appearance of the product (Fig. 1e), the ICP data, and the disappearance of green phosphorescence after the acid treatment (Fig. S1†) highly indicate that the immersion of the raw ground EX-Kaol in an aqueous solution containing Tb^3+^ ions caused these ions to be adsorbed on the kaolinite edge surfaces, which can function as cation exchange sites.^10,11^ The 0.12 mol% Tb_2_O_3_ concentration in the m-CAS-Tb-Edge is a reasonable result because this value is smaller than the maximum Tb_2_O_3_ content of 0.32 mol% estimated based on the cation exchange capacity of pristine kaolinite (2.3 mmol/100 g clay).^11^ In addition, the Tb^3+^ adsorbed on the edges likely formed inner sphere complexes according to previous reports.^27,28^ It is therefore probable that Tb^3+^ can be effectively incorporated into metastable CaAl_2_Si_2_O_8_ by adsorption on the kaolinite edges.

Because the XRD reflections generated by the metastable CaAl_2_Si_2_O_8_ were originally broad, changes in the positions of these reflections were difficult to detect (Fig. 3). Since rare-earth ions can also form outer sphere complexes on kaolinite layer surfaces,^27,28^ the Tb^3+^ in this material could have been surrounded by water molecules and converted into Tb_2_O_3_ on the m-CAS-Tb-Edge surfaces, as has been proposed in previous reports.^24–28^ The XRD pattern (Fig. 3f) and Tb_2_O_3_ concentration (0.12 mol %^−1^) for m-CAS-Tb-Edge together with the relatively weak luminescence from the original Tb_2_O_3_ (Fig. 1b) demonstrate that Tb_2_O_3_ would have made only a minor contribution to the green luminescence from m-CAS-Tb-Edge. Additionally, although anorthite is a mother material for Tb^3+^-containing phosphors,^31,32^ the relatively weak reflection attributed to anorthite (Fig. 3f) indicates that the green luminescence from m-CAS-Tb-Edge was due to the formation of Tb^3+^-doped metastable CaAl_2_Si_2_O_8._ In our previous studies,^7,9^ the kaolinite grinding process was found to generate new hydroxyl groups by breaking Al–O–Si bonds^7^ and the expanded kaolinite increased the available edge surfaces.^9^ The present immersion method could therefore be an effective means of doping the ground mixture of Ex-Kaol and CaCO_3_. Because the ground raw material exhibited some green phosphorescence after immersion in the Tb^3+^ solution (Fig. S1,† left), there may have been limited adsorption of Tb^3+^ on the CaCO_3_ surface. Notably, the effect of the kaolinite edges was also confirmed by the results obtained with the present m-CAS-Ca-Edge (Fig. 4), which exhibited more rapid formation of anorthite compared with m-CAS. This result suggests that the formation of this stable phase was promoted by Ca^2+^ ions that were possibly present on the kaolinite edges. In contrast to m-CAS, those materials containing Tb^3+^ slowly formed metastable CaAl_2_Si_2_O_8_ along with an increase in byproduct formation (see Fig. 1a–f and the experimental procedures). In this case, Tb^3+^ can be regarded as an impurity that suppressed the rapid formation of metastable CaAl_2_Si_2_O_8_, in good agreement with our previous result.^9^ However, further study will be required to clarify the detailed mechanisms associated with the effects of kaolinite edges on which various cations are adsorbed.

**Fig. 5:**
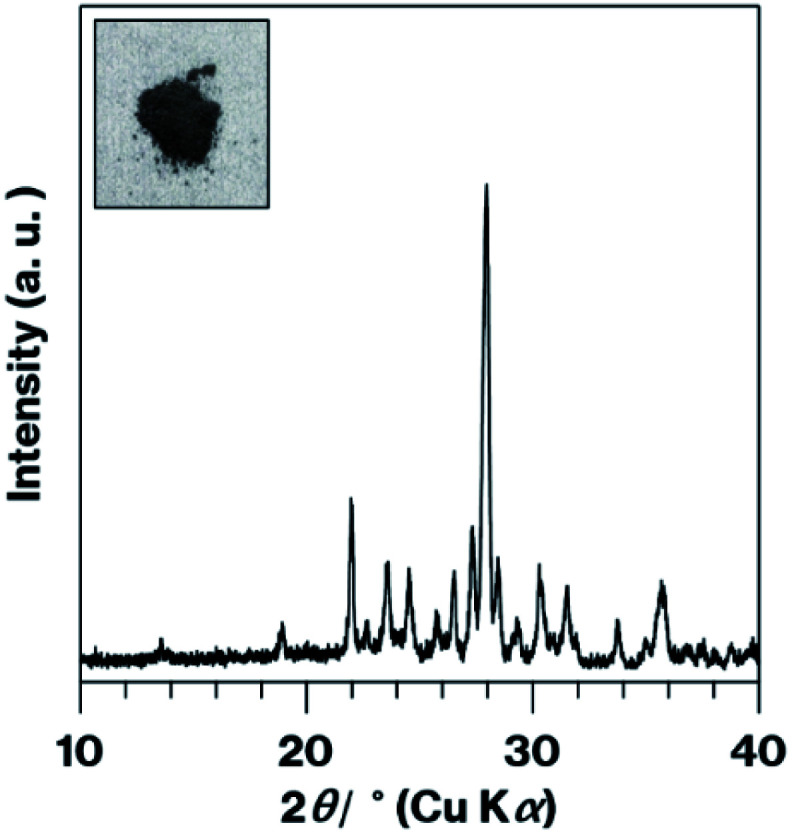
XRD pattern and photographic image (inset) for the m-CAS-Ca-Edge.

Interestingly, m-CAS-R was black in colour and produced an XRD pattern containing anorthite reflections (Fig. 5). It is assumed that the solid-state reaction of kaolinite and CaCO_3_ together with various Tb^3+^ compounds inhibited the formation of a metastable CaAl_2_Si_2_O_8_-based phosphor under a reductive atmosphere. The action of the kaolinite edges is therefore necessary for the formation of a green phosphor *via* a solid-state reaction under air. It should also be noted that grinding of kaolinite nanoscrolls to form nanoparticles^33^ could potentially increase the edge surface area. The present Tb^3+^-doped ground materials are likely similar to Tb^3+^-containing substances previously prepared by sol–gel reactions, some of which could be calcined to form phosphors under a reductive atmosphere.^34^ To the best of our knowledge, there have been few reports of the colour of such materials in powder form after preparation based on a solid-state reaction. The present study clearly demonstrates the formation of a material that emits green light in response to excitation by the solid-state reaction of Tb^3+^-adsorbed raw materials under air.

## Conclusions

We have demonstrated the effect of kaolinite edges on a solid-state reaction. Specifically, the incorporation of Tb^3+^ in metastable CaAl_2_Si_2_O_8_ was accomplished using ground raw materials containing kaolinite with Tb^3+^ adsorbed on its edge surfaces. The resulting product could be useful not only as a phosphor but also as a composite material.^22^ Furthermore, the present method could be applied to promote other solid-state reactions of cation-exchangeable layered inorganic solids.^35^

## Author contributions

Shingo Machida: conceptualization, data curation, investigation, writing—original draft, supervision. Ken-ichi Katsumata: writing—review and editing. Atsuo Yasumori: project administration.

## Conflicts of interest

There are no conflicts to declare.

## Supplementary Material

RA-012-D2RA02199D-s001
