# Supplementary material for: Effect of kaolinite edge surfaces on formation of Tb3+-doped phosphor by solid-state reaction
Source: RSC Adv. 2022 May 20;12(24):15435–9. doi: 10.1039/d2ra02199d (PMC9121353; doi:10.1039/d2ra02199d)
Supplement: RA-012-D2RA02199D-s001 [file RA-012-D2RA02199D-s001.pdf]

Electronic Supplementary Material (ESI) for RSC Advances.
This journal is © The Royal Society of Chemistry 2022

Electronic Supporting Information

Effect of kaolinite edge surfaces on formation of Tb^{3+}-doped phosphor by solid-state reaction

Shingo Machida, Ken-ichi Katsumata, and Atsuo Yasumori*

Department of Material Science and Technology, Faculty of Advanced Engineering, Tokyo University of Science, 6-3-1 Niijuku, Katsushika-ku, Tokyo 125-8585, Japan

*E-mail: shingo.machida@rs.tus.ac.jp

Figures

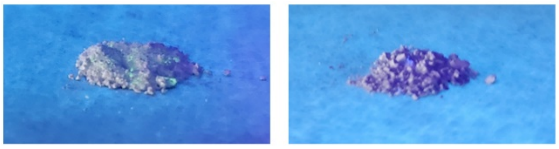

Figure S1. Photograph of the Tb^{3+} solution-immersed ground raw materials before and after the acid treatment (left to right).

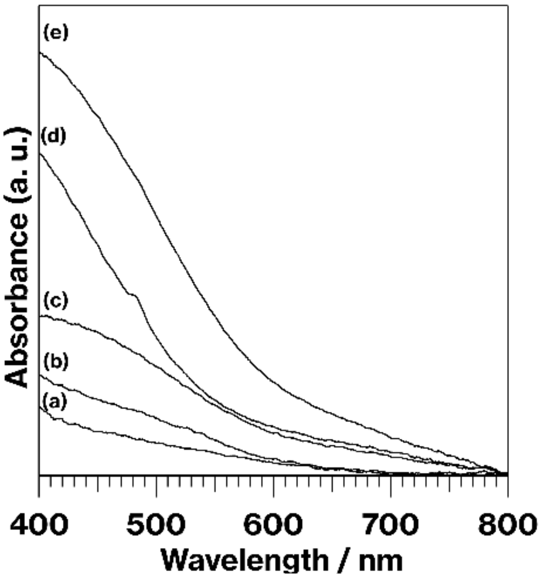

Figure S2. Visible-light spectra of (a) m-CAS, (b) m-CAS-Tb-Edge, (c) m-CAS-Tb-0.075, (d) Tb_2O_3, and (e) m-CAS-Tb-0.3.

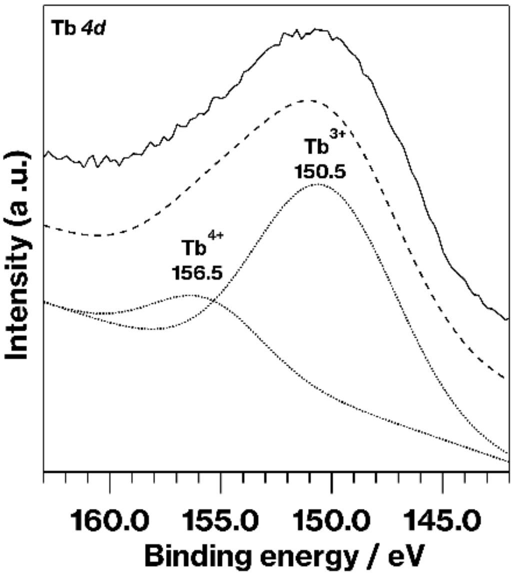

Figure S3. XPS spectrum of Tb_2O_3 (solid line). Dotted lines and dashed line are deconvolutional components and simulation spectrum, respectively.

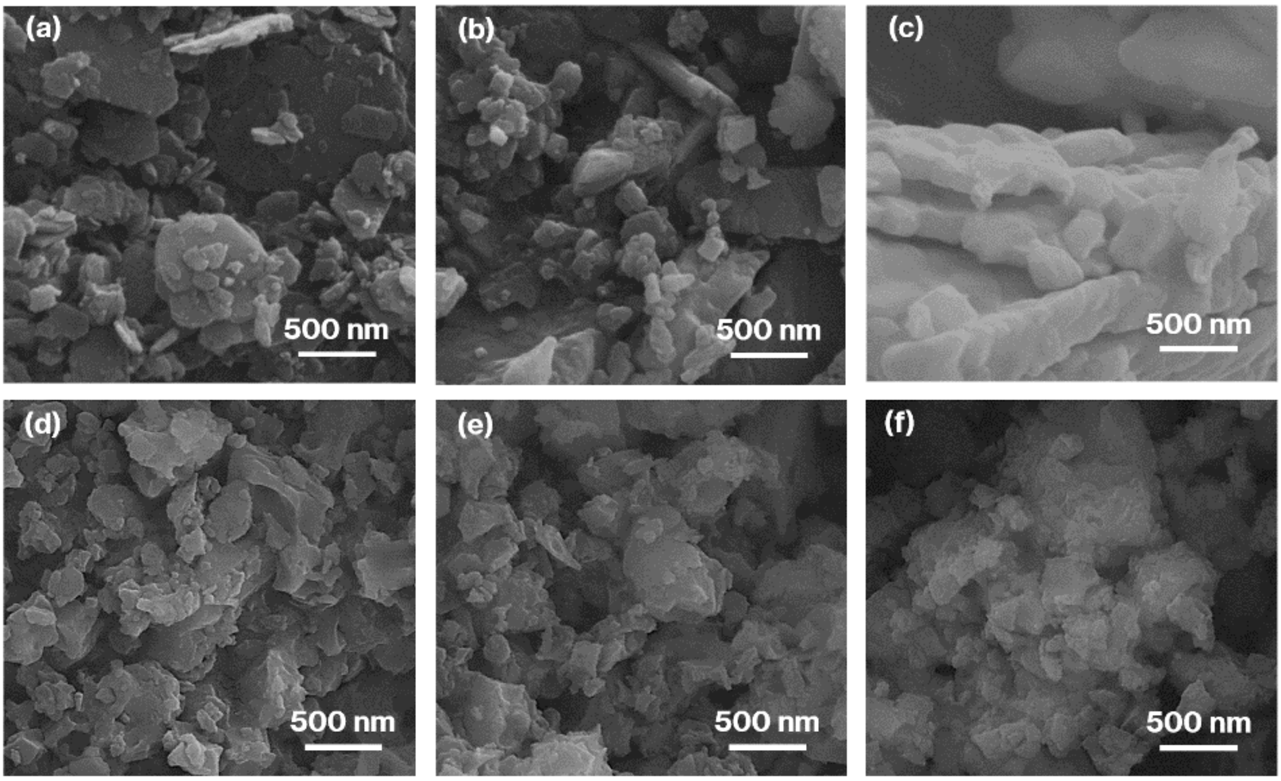

Figure S4. FE-SEM images of (a) Ex-Kaol, (b) $CaCO_3$, (c) Tb_2O_3, (d) m-CAS, (e) m-CAS-Tb-0.3, and (f) m-CAS-Tb-Edge.

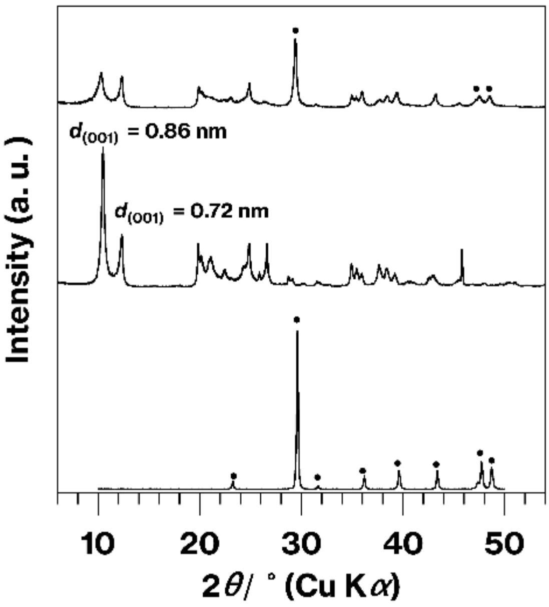

Figure S5. XRD patterns for $CaCO_3$, Ex-Kaol, and their ground raw materials (from bottom to top). The filled circles indicate calcite reflections.
